# Complete genome sequence of producer of the glycopeptide antibiotic Aculeximycin *Kutzneria albida* DSM 43870^T^, a representative of minor genus of *Pseudonocardiaceae*

**DOI:** 10.1186/1471-2164-15-885

**Published:** 2014-10-10

**Authors:** Yuriy Rebets, Bogdan Tokovenko, Igor Lushchyk, Christian Rückert, Nestor Zaburannyi, Andreas Bechthold, Jörn Kalinowski, Andriy Luzhetskyy

**Affiliations:** Helmholtz-Institute for Pharmaceutical Research Saarland, Saarland University Campus, Building C2.3, 66123 Saarbrücken, Germany; Center for Biotechnology, Bielefeld University, Universitätsstraße 27, 33615 Bielefeld, Germany; Institut für Pharmazeutische Biologie und Biotechnologie, Albert-Ludwigs Universität, Stefan-Meier-Strasse 19, 79104 Freiburg, Germany

**Keywords:** Kutzneria, Genome, Genomic islands, Secondary metabolism, Aculeximycin

## Abstract

**Background:**

*Kutzneria* is a representative of a rarely observed genus of the family *Pseudonocardiaceae. Kutzneria* species were initially placed in the *Streptosporangiaceae* genus and later reconsidered to be an independent genus of the *Pseudonocardiaceae. Kutzneria albida* is one of the eight known members of the genus. This strain is a unique producer of the glycosylated polyole macrolide aculeximycin which is active against both bacteria and fungi. *Kutzneria albida* genome sequencing and analysis allow a deeper understanding of evolution of this genus of *Pseudonocardiaceae*, provide new insight in the phylogeny of the genus, as well as decipher the hidden secondary metabolic potential of these rare actinobacteria.

**Results:**

To explore the biosynthetic potential of *Kutzneria albida* to its full extent, the complete genome was sequenced. With a size of 9,874,926 bp, coding for 8,822 genes, it stands alongside other *Pseudonocardiaceae* with large circular genomes. Genome analysis revealed 46 gene clusters potentially encoding secondary metabolite biosynthesis pathways. Two large genomic islands were identified, containing regions most enriched with secondary metabolism gene clusters. Large parts of this secondary metabolism “clustome” are dedicated to siderophores production.

**Conclusions:**

*Kutzneria albida* is the first species of the genus *Kutzneria* with a completely sequenced genome. Genome sequencing allowed identifying the gene cluster responsible for the biosynthesis of aculeximycin, one of the largest known oligosaccharide-macrolide antibiotics. Moreover, the genome revealed 45 additional putative secondary metabolite gene clusters, suggesting a huge biosynthetic potential, which makes *Kutzneria albida* a very rich source of natural products. Comparison of the *Kutzneria albida* genome to genomes of other actinobacteria clearly shows its close relations with *Pseudonocardiaceae* in line with the taxonomic position of the genus.

**Electronic supplementary material:**

The online version of this article (doi:10.1186/1471-2164-15-885) contains supplementary material, which is available to authorized users.

## Background

Bacteria of the *Actinomycetales* order represent an amazing source of biologically active compounds which are produced as secondary metabolites [1]. The broad spectrum of structural features and wide array of activities of these metabolites attract attention as a limitless source of novel chemical scaffolds as well as new drugs for human and veterinary medicine, and agriculture. With the recent advances in sequencing technologies significant progress in sequencing and analysis of actinobacterial genomes was achieved [2, 3]. Not only genomes of biotechnologically important and typical representatives of the taxonomical order were sequenced, but also strains with interesting and unusual features, leading to deeper insights into phylogeny, genetics, physiology, ecology, and the secondary metabolism biosynthetic potential of these bacteria. One of the major discoveries of the genomic era in actinomycetes research is the presence of multiple gene clusters dedicated to secondary metabolism in one genome. This observation caused an increase of interest in diverse groups of actinomycetes (especially rare representatives of its subgroups) as a possible source of new biologically active metabolites. One of such groups includes species belonging to the *Pseudonocardiaceae* family*.* Multiple genome projects dedicated to this group of bacteria are in progress, with seven being completed and published [4, 5]. Here we report the sequencing and analysis of the complete genome of *Kutzneria albida* DSM 43870^T^ (formerly “*Streptosporangium albidum”*) [6, 7]. The genus *Kutzneria* is a minor branch of the *Pseudonocardiaceae* family currently containing only 8 species [8–10]. To our knowledge, this is the first member of the *Kutzneria* genus and only the eighth representative of the family *Pseudonocardiaceae* for which a complete genome sequence is available.

*K albida* was isolated from soil samples collected in the rocky regions of Gunma Prefecture of Japan [6]. The strain is known as a producer of aculeximycin, a macrolide antibiotic with an interesting chemical structure and activity against Gram-positive bacteria, fungi, and mosquito larvae [11, 12]. Being generally toxic, aculeximycin cannot be used in medicine [13]. However, some structural features, especially the unusual glycoside chain, make it an attractive source of building blocks for use in synthetic biotechnology and combinatorial biosynthesis of secondary metabolites [14, 15]. Sequencing *K. albida* genome allowed not only to identify genes responsible for aculeximycin biosynthesis, but also to discover an unusual abundance and diversity of secondary metabolites that could potentially be produced by this species. To the best of our knowledge, this genome is the most enriched with clusters involved in secondary metabolites among actinobacterial genomes.

## Results and discussion

### General features of the genome

**Figure 1:**
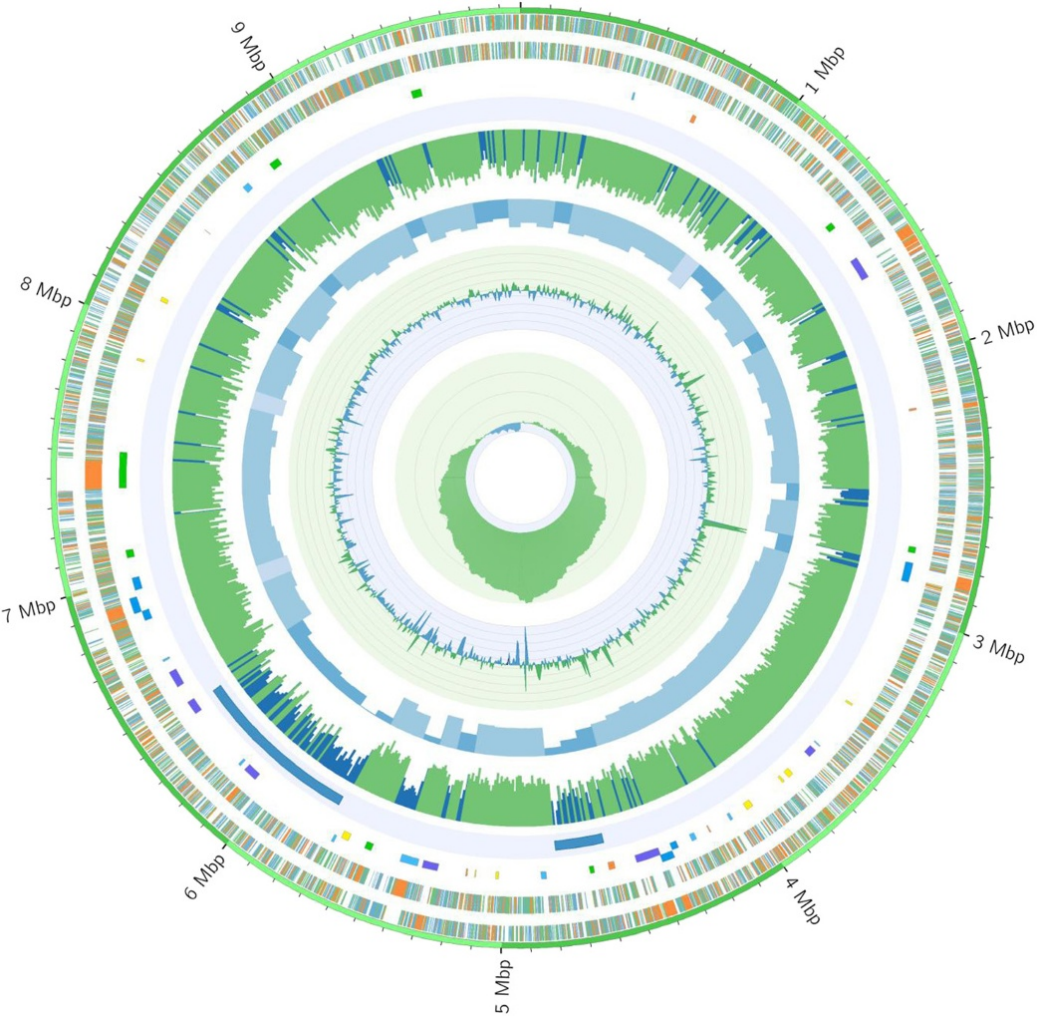
**Schematic representation of the** ***Kutzneria albida*** **genome, created with the help of Circos [**[19]**]; megabases are labeled; smaller ticks correspond to 100 kbp segments.** From outside: genes on the forward and the reverse strands (blue: shorter than 900 bp, green: between 900 and 1500 bp long, orange: longer than 1500 bp); 46 secondary metabolite clusters coloured by type (NRPS: blue; siderophore NRPS: lighter blue; PKS: green; hybrid PKS-NRPS: dark purple; terpene: orange; other: yellow); genomic islands; G + C content, 10kbp window (blue colour highlights segments with G + C content <69%); G + C content, 100 kbp window (lighter blue is higher G + C, darker blue is lower G + C content); G + C skew (green: positive; blue: negative); cumulative G + C skew. The *oriC* is placed at coordinate zero.

After gaps closure, a single contig with the size of 9,874,926 bp and a 70.6% G + C content was obtained. General features of *K. albida* genome are summarized in Table 1. The genome of *K. albida* consists of a single circular replicon; no extrachromosomal replicon was detected. The origin of replication (*oriC*) was identified as a 1,044 nt non-coding region between the two genes – *dnaA*, encoding the chromosomal replication initiation factor, and *dnaN*, coding for the β-subunit of the DNA polymerase III (Figure 1). Interestingly, the *oriC* has no classic DnaA boxes [TT(G/A)TCCACA], and shows almost no similarity to the *oriC* of *Streptomyces* species [16, 17] *.* On the opposite side of the chromosome from *oriC*, a putative *dif* (*deletion-induced filamentation*, chromosome resolving site) locus was identified. Its sequence is in good accordance with actinobacterial *dif* sites [18]. Additionally, the obvious cumulative GC skew inversion in the regions of *oriC* and *dif* supports our *oriC* assignment and suggests the existence of two replichores composing the circular *K. albida* chromosome (Figure 1).

**Table 1:** **General features of the** ***K. albida*** **chromosome**

Type	Property
**Topology**	Circular
**Number of replicons**	1
**Total size**	9,874,926
**G + C content**	70.6%
**Protein coding genes**	8,822
**rRNA genes**	9
**rRNA operons**	3
**Transfer RNAs**	47 (19 species)
**Average gene length**	981
**Coding density**	88.49%
**Secondary metabolite clusters**	46

Unlike the linear topology of *Streptomyces* genomes, the chromosome of *K. albida* is circular (Table 1) [20]. This appears to be a common feature of *Pseudonocardiaceae* representatives genomes, since the chromosomes of other representatives of this family sequenced so far also have a circular topology [5]. With a total length of 9,874,926 bp, the *K. albida* chromosome is larger than that of *Streptomyces coelicolor* A3(2) (8.7 Mbp) [21] and *Streptomyces avermitilis* MA-4680 (9.0 Mbp) [22], as well as genomes of *Pseudonocardiaceae Saccharomonospora viridis* P101^T^ (4.3 Mbp) [23], *Pseudonocardia dioxanivorans* CB1190 (7.1 Mbp) [24], *Saccharothrix espanaensis* DSM 44229^T^ (9.3 Mbp) [5], and *Saccharopolyspora erythraea* NRRL 2338 (8.2 Mbp) [25], but slightly smaller than the genome of another *Pseudonocardiaceae* rifamycin producer *Amycolatopsis mediterranei* S699 (10.2 Mbp) [26]. The *K. albida* genome is among the largest actinobacterial genomes sequenced so far.

*K. albida* chromosome contains 8,822 predicted protein-coding sequences (CDSs) (Table 1). Three ribosomal RNA (*rrn*) operons were identified in the genome along with 47 tRNA genes (Table 1) [27]. The *rrn* operons are located on the leading strand of the chromosome proximally to *oriC* and have remarkably low G + C content in comparison to the genome average.

Functional annotation of *K. albida* genes within the actNOG (Actinobacteria) subset of the eggNOG database (using protein BLAST with an expectation value cut-off 0.001) [28], showed that 6,648 (75%) out of 8,822 genes had at least one biological function assigned (Table 2), with some of the genes assigned to more than one category. Of the remainder, 960 CDSs (10.8%) had no hits against actNOGs, and 1,591 genes (18%) had hits but were not assigned to functional categories; of the latter, 47 are highly conserved. Among the genes with functional assignment, 2,493 (28%) are implicated in metabolism, including 403 (4.5%) in amino acid metabolism, 519 (5.8%) in carbohydrate metabolism, and 295 (3.3%) participating in secondary metabolism (Table 2). At the same time, 10% of the annotated genes (836) code for proteins involved in transcriptional processes, including 60 sigma factors, 15 anti-sigma factors, 6 anti-anti-sigma factors, and 747 (8.4%) putative DNA binding regulators. The large number of proteins implicated in transcriptional processes suggests a high complexity of *K. albida* gene expression control. The high proportion of proteins involved in transcriptional regulation is accompanied by a similarly high percentage of proteins involved in signal transduction pathways (350 genes, 4%), which suggests a close connection between extracellular nutrient sensing and transcriptional regulation of uptake and degradation pathways. This feature of the *K. albida* genome is typical for the majority of known soil living actinobacteria and reflects their life style and ecological niche, providing higher flexibility of the strain adaptation to changes in growth conditions [20]. In addition to this, a large portion of the genome encodes extracellular polymer-degrading enzymes. In total, 94 putative secretory hydrolase encoding genes were found in the *K. albida* genome (1% of all genes), including 18 glycoside hydrolases and 20 extracellular proteases.

**Table 2:** **Assignment of 6,648** ***K. albida*** **genes to the functional groups of the actNOG subset of the eggNOG database**

**Information storage and processing**	**1,302**
**Translation, ribosomal structure and biogenesis**	182
**RNA processing and modification**	1
**Transcription**	836
**Replication, recombination and repair**	282
**Chromatin structure and dynamics**	1
**Cellular processes and signaling**	936
**Cell cycle control, cell division, chromosome partitioning**	35
**Nuclear structure**	0
**Defense mechanisms**	116
**Signal transduction mechanisms**	350
**Cell wall/membrane/envelope biogenesis**	214
**Cell motility**	2
**Cytoskeleton**	2
**Extracellular structures**	0
**Intracellular trafficking, secretion, and vesicular transport**	27
**Posttranslational modification, protein turnover, chaperones**	190
**Metabolism**	2,493
**Energy production and conversion**	469
**Carbohydrate transport and metabolism**	403
**Amino acid transport and metabolism**	519
**Nucleotide transport and metabolism**	99
**Coenzyme transport and metabolism**	179
**Lipid transport and metabolism**	291
**Inorganic ion transport and metabolism**	238
**Secondary metabolites biosynthesis, transport and catabolism**	295
**Poorly characterized**	2,192
**General function prediction only**	601
**Function unknown**	1,591

A region of the chromosome (approximately 4 Mbp) extending either side of *oriC* appears to contain the majority of the genes predicted to be essential with the highest density in close proximity to *oriC* (Figure 1). This distribution of housekeeping genes with the notable core region is typical for actinobacterial genomes [5, 21, 25]. Outside this region, the chromosome has apparently undergone major expansion by acquiring novel genetic elements as a result of horizontal gene transfer. This *oriC*-distal region is enriched with the genetic loci encoding secondary metabolism, many of which have little similarity to other *Pseudonocardiaceae* secondary metabolism genes. After manual correction of AntiSMASH results [29], approximately 14% of the *K. albida* chromosome (1.46 Mbp) was found to be involved in secondary metabolites biosynthesis. This includes 852 ORFs, or 9.6% of all genes annotated in the genome. Thus, secondary metabolism genes occupy a significantly bigger proportion of *K. albida* genome when compared to other actinomycetes, including sequenced genomes of *Pseudonocardiaceae*. For example, *S. coelicolor* A3(2) secondary metabolism genes cover only 5% of the chromosome, whereas in *S. avermitilis* MA-4680 secondary metabolism genes represent 6.6% of the genome length [22].

Another feature of the *K. albida* chromosome is the presence of numerous genomic islands outside the ancestral core genome. These regions comprise the youngest part of the chromosome acquired as a result of horizontal gene transfer events. They are characterized by lower than average genomic G + C content, biased codon usage, as well as presence of numerous insertion elements and transposon relicts. Among *K. albida* genomic islands, the most interesting are the two large regions with a length of 204 kbp (4583813–4788042) and 718 kbp (5733548–6452132), respectively (Figure 1). These regions are significantly distinct from the rest of the genome. Additional synteny analysis between *K. albida* and draft genome sequence of *K. spp.* 744 (SRX005446, SRX005562) clearly showed that the longer genomic island is acquired [30]. We were unable to determine the origin of the 204 kbp genomic island, but in the case of the 718 kbp region there were clearly no counterparts identified in sequence of *K. spp.* 744 genome, even though the regions around this portion of the *K. albida* chromosome are similar in both species. Both regions designated as genomic island are surrounded by the long inverted repeats (432 bp, 69% identical bp in the case of 204 kbp island, and 323 bp, 70% identical bp in the case of 718 kbp island) that might point on their origin from the large plasmids that were inserted into the chromosome. However, other possibilities cannot be excluded. Interestingly, the location of the predicted genomic islands corresponds to the locations of the regions with high density of secondary metabolism gene clusters indicating their possible origin (Figure 1).

### Phylogenetic and orthologous analysis

**Figure 2:**
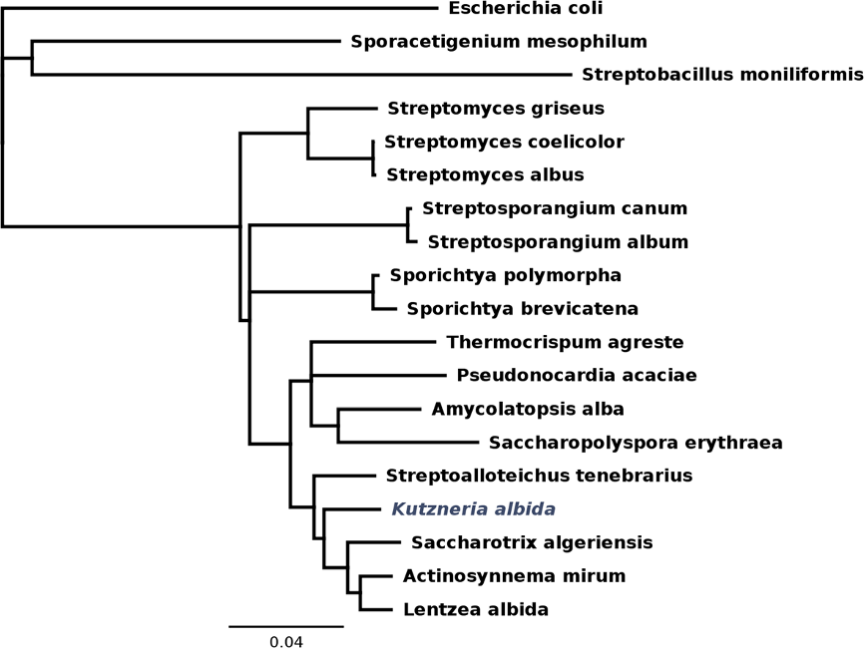
**The 16S rRNA phylogram of representative** ***Actinobacteria*** **strains, with an outgroup containing E. coli as non-related strains.** Ribosomal RNA sequences for all strains but *S. albus* were obtained from the Silva rRNA database [32]. Bootstrap value 1000.

*K. albida* was originally classified as “*Streptosporangium albidum”* in 1967 [6]. However, later it was renamed to *Kutzneria albida* due to the primary structures of 5S rRNA and 16 s rRNA, as well as to chemotaxonomic properties of the strain and the electrophoretic mobility of its ribosomal proteins [10]. Consequently, the genus was transferred to the family *Pseudonocardiaceae*, due to the range of taxonomic features that were found to be more similar to the typical strain of the family, *Saccharothrix australiensis* ATCC 31497^T^ [10, 31]. An unsupervised nucleotide BLAST analysis of the DNA sequence corresponding to the 16S rRNA gene from *K. albida* with the 16S rRNA of different actinobacteria and *E. coli* as out-group was performed to determine phylogenetic relationships of the strain within the taxon (Figure 2). This analysis clearly showed that *K. albida* is distinct from the genus *Streptosporangium* and *Streptomyces,* and closer related to representatives of *Pseudonocardiaceae*. The highest similarity was observed between 16S rRNA of *K. albida* and *Lentzea albida, Actinosynnema mirum, Saccharothrix algeriensis, Streptoalloteichus tenebrarius, Saccharopolyspora erythraea* and *Amycolatopsys alba*, all of which belong to the *Pseudonocardiacea* family. At the same time, it is clear that the closest relatives of *K. albida* inside *Pseudonocardiaceae* are *Streptoalloteichus tenebrarius, Lentzea albida*, *Actinosynnema mirum*, and *Saccharothrix algeriensis* (Figure 2)*.* These species are forming a branch distinct from other representatives of the family.

The molecular phylogeny fully corresponds to and supports the data obtained during comparison of 16S rRNA. As expected, *K. albida* shares the highest number of orthologous genes (reciprocal best-hit pairs in pairwise protein BLAST searches) with the *Pseudonocardiaceae* and less with the *Streptosporangiaceae* (Table 3)*.* Ten genomes of actinobacteria were used in this analysis: *Kutzneria albida* DSM 43870^T^, *Actinosynnema mirum DSM 43827*^T^*, Amycolatopsis mediterranei S699, Kitasatospora setae* NBRC 14216^T^*, Saccharopolyspora erythrea NRRL 2338, Saccharotrix espanaensis DSM 44229*^T^*, Streptomyces avermitilis MA-4680, Streptomyces coelicolor A3(2), Streptomyces griseus subsp. griseus NBRC 13350, Streptosporangium roseum DSM 43021*^T^*.* As expected, *K. albida* shares the highest number of orthologs with *A. mediterranei* (3,726), followed by *Saccharotrix espanaensis* (3,585 genes), *Actinosynnema mirum,* and *S. erythraea* (3,311 and 3,260 genes, respectively). In comparison, the number of genes shared with *Streptosporangium roseum* is 3,144. In contrast, the *K. albida* genome shares less orthologous genes with all the species from the genus *Streptomyces* tested: *S. coelicolor* – 3,017, *S. avermitilis* – 3,031, *S. griseus* – 2,935. Furthermore, when comparing all analyzed genomes, the number of orthologs drops to 1,766 genes, defining the non-strict minimal core genome shared between the analyzed species. This number is bigger than the previously predicted core genome for the family [5] because of a less strict method of calculating orthologs used in this study. Our result should not be regarded as a disagreement with earlier results, because our goal is only to provide additional support for the proper placement of *Kutzneria albida* among other *Pseudonocardiaceae* – we are not aiming at the identification of the 10-species core genome. Most of these 1,766 genes are located around *oriC*, while genes unique to *K. albida* or conserved in only one species are located further away from the *oriC*.

**Table 3:** **Number of orthologous genes shared by** ***K. albida*** **and each one of the other 9** ***Actinomycetales*** **genomes**

Strain	1	2	3	4	5	6	7	8	9	10
1. *Saccharotrix espanaensis*		3640	3556	3098	3143	3585	2940	2882	2930	2827
2. *Actinosynnema mirum*			3328	2840	2964	3311	2855	2754	2756	2622
3. *Amycolatopis mediterranei*				3222	3249	3726	3138	3121	2953	2858
4. *Streptosporangium roseum*					2885	3144	3151	3126	3102	2994
5. *Saccharopolispora erythraea*						3260	2833	2781	2745	2563
6. *Kutzneria albida*							3017	3031	2935	2841
7. *Streptomyces coelicolor*								4013	3889	3352
8. *Streptomyces avermitilis*									3753	3327
9. *Streptomyces griseus*										3344
10. *Kitasatospora setae*										
All strains										1766

Last row shows orthologs common to all 10 genomes.

**Table 4:** **Comparison of the secondary metabolome of different actinobacteria**

Species	Genome size	Terpene	PKS	NRPS	Hybrid	Other	Total
***S. espanaensis*** **DSM 44229** ^**T**^	9 360 653	7	3	5	6	5	26
***A. mirum*** **DSM 43827** ^**T**^	8 248 144	4	10	3	5	1	23
***A. mediterranei*** **S699**	10 246 920	4	6	7	4	4	25
***S. roseum DSM 43021*** ^**T**^	10 341 314	5	5	9	1	6	26
***S. erythraea*** **NRRL 2338**	8 212 805	10	11	4	2	3	30
***K. albida*** **DSM 43870** ^**T**^	**9 874 926**	**5**	**8**	**16**	**7**	**10**	**46**
***S. coelicolor*** **A3(2)**	8 667 507	4	7	3	1	9	24
***S. avermitilis*** **MA-4680**	9 025 608	6	11	8	2	11	38
***S. griseus*** **NBRC 13350**	8 545 929	8	6	7	5	8	34
***K. setae*** **NBRC 14216** ^**T**^	8 783 278	5	4	5	3	7	24
***S. viridis*** **P101** ^**T**^	4 308 349	2	2	2	0	2	8
***P. dioxanivorans*** **CB1190**	7 096 571	2	0	1	0	2	5

### Aculeximycin biosynthesis gene cluster

**Figure 3:**
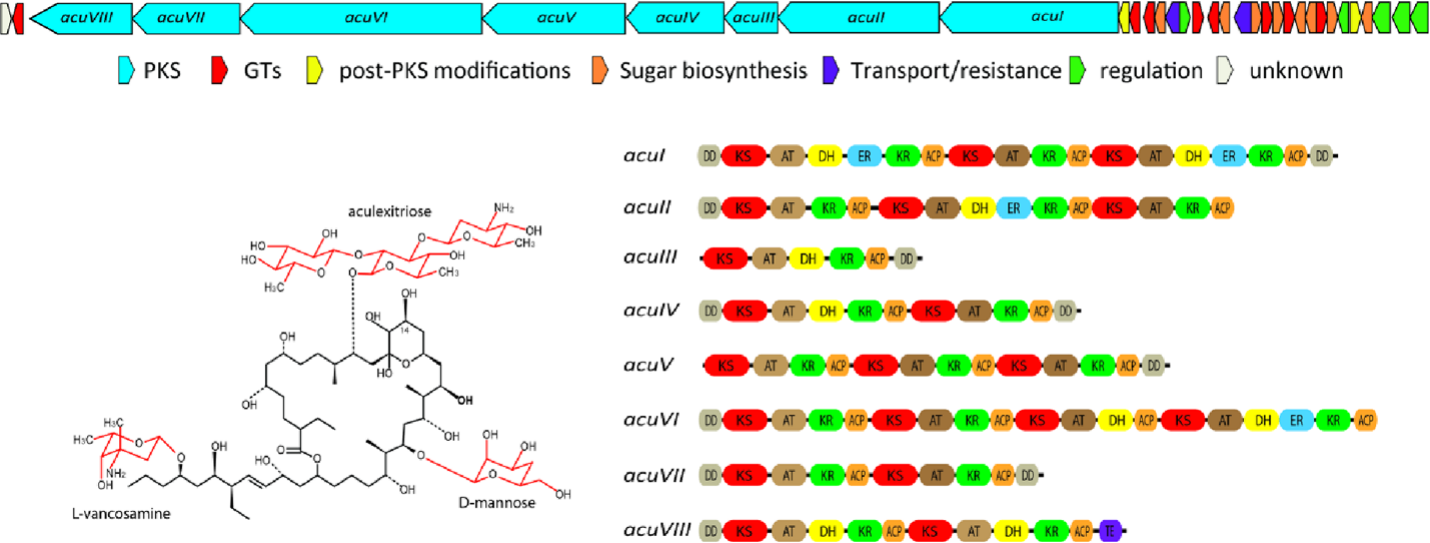
**Chemical structure of aculeximycin and genetic organization of the** ***acu*** **biosynthesis gene cluster.** PKS domain prediction was performed with antiSMASH and SEARCHPKS tools [29, 35]. Domain abbreviations: DD – docking domain, KS – ketosynthase, AT – acyltransferase, DH – dehydratase, ER – enoylreductase, KR – ketoreductase, ACP – acyl carrying protein, TE – thioesterase.

The only characterized secondary metabolite produced by *K. albida* is aculeximycin (Figure 3) [12, 15]. This compound is particularly interesting due to its activity against a broad range of Gram-positive bacteria, as well as against fungi and mosquitoes [12]. Aculeximycin, like similar metabolite streptoviridin from *Kutzneria viridogrisea*, exerts strong general toxicity caused by uncoupling of oxidative phosphorylation in mitochondria [13, 33]. On the other hand, this compound has an intriguing chemical structure with five sugars attached to the macrolactone [14, 15, 34]. Analysis of the *K. albida* genome sequence revealed the entire set of genes required for the aculeximycin molecule assembly clustered in a region of the chromosome approximately 2.3 Mbp away from the *oriC*. The *acu* cluster is 141.5 kbp long and contains 34 ORFs (Figure 3; all *acu* genes with predicted function are listed in Additional file 1: Table S1). The core of the cluster comprises eight genes encoding type I polyketide synthases (ORFs KALB_6560 – KALB_6567) with 21 modules in total, each containing different sets of reductase and acyltransferase domains as predicted by antiSMASH and SEARCHPKS (Figure 3, Additional file 1: Table S1) [29, 35]. Since biosynthesis of the aculeximycin polyketide scaffold is predicted to require 20 condensation steps including loading it makes us believe that one module is skipped during elongation process or alternate utilization of two modules takes place [36]. The first module encoded by *AcuAI* (KALB_6567), a three-modular synthase, contains a full set of ketoreduction domains that corresponds to the first condensation step of biosynthesis. However, neither the loading module nor the other four first elongation steps could be predicted based on a collinear logic of polyketide assembly, since the sets of ketoreduction domains in the second and third modules of *AcuAI* (KALB_6567) and three modules of *AcuAII* (KALB_6566) did not correspond to the primary structure of the aculeximycin polyketide [15, 37]. The last module encoded by *acuAVIII* (KALB_6560), contains a thioesterase domain (TE) similar to TE domains of erythromycin and pikromycin synthases that are catalysing lactonization of the matured chain [38, 39]. Malonate, methylmalonate, and ethylmalonate are used as precursors in polyketide chain extension. The hydroxyl group at C14 position is most probably incorporated as post-PKS oxygenation, and the gene encoding a hydroxylase, *acuO2* (KALB_6568), is present within the cluster (Figure 3, Additional file 1: Table S1).

Aculeximycin is a highly glycosylated compound (Figure 3). Two sugars – D-mannose and L-vancosamine – are attached at positions C23 and C37, respectively [15, 34]. A short oligosaccharide chain named aculexitriose (O-6-deoxy-beta-D-glucopyranosyl-(1–2)-O-[3-amino-2,3,6-trideoxy-beta-D-arabino-hexopyranosyl-(1–3)]-6-deoxy-D-glucopyranose) is attached at position C11. D-mannose is supplied from the primary metabolism, as in the case of biosynthesis of other antibiotics containing this moiety [40]. Deoxysugar biosynthesis genes are present within the cluster: The genes *acuS6* (KALB_6571) and *acuS2* (KALB_6585) encode glucose-1-phosphate thymidylyltransferase and dTDP-glucose 4,6-dehydratase, respectively – enzymes participating in the initial common steps in deoxysugars biosynthesis [41, 42]. Genes for downstream enzymes leading to the production of each individual sugar from NDP-4-keto-6-deoxy-D-glucose are also present within the cluster. L-vancosamine is built by the sequential action of *AcuS1* (NDP-hexose-C3-methyltransferase, KALB_6588), *AcuS3* (NDP-4-dehydrorhamnose-3,5-epimerase, OF6583), *AcuS4* (NDP-hexose-2,3-dehydratase, KALB_6582), *AcuS5* (NDP-hexose-4-ketoreductase, KALB_6576), and *AcuN1* (KALB_6580) or *AcuN2* (KALB_6578) aminotransferases, similar to what had been described for the glycopeptide antibiotic chloroeremomycin [43]; 6-deoxy-glucopyranose – *AcuS5*; 3-amino-2,3,6-trideoxy-beta-D-arabino-hexopyranose – *AcuS4*, *AcuS5*, *AcuN1* or *AcuN2* (Additional file 1: Table S1). Eight putative glycosyltransferase genes (*acuGT1-8*; KALB_6584, 6581, 6579, 6575, 6574, 6570, 6569, 6559) were found within the aculeximycin biosynthesis gene cluster. The redundant number of GTs can be explained by the fact that some of them might participate in self-resistance mechanism. *AcuGT3* (KALB_6579) has a high degree of similarity to numerous glycosyltransferases involved in resistance to macrolides, including one involved in self-resistance of oleandomycin producer [44, 45]. Another component of this resistance complex might be the gene product of *acuH* (KALB_6577) encoding a β-hexosaminidase homologue. This family of enzymes is involved in the cleavage of GalNAc residues from oligosaccharides. The *acu* cluster also contains *acuW* (KALB_6572) gene encoding type III ABC transporter protein containing both transmembrane and ATP binding domains within one polypeptide [46, 47]. In addition, 5 putative regulatory genes were found within the *acu* cluster. One of them, *AcuR5* (KALB_6573), belongs to the TetR family of transcription regulators and is located next to the *acuW* gene. Four other regulators (*AcuR1-4*: OF6591, 6590, 6589, 6586) belong to the LuxR family and might participate in control and fine tuning of the aculeximycin production (Figure 3, Additional file 1: Table S1) [48].

### Secondary metabolites production potential

Besides aculeximycin, no other secondary metabolites are known to be produced by *K. albida*. However, genome analysis using antiSMASH revealed 47 gene clusters potentially involved in secondary metabolism, including the *acu* cluster [29]. Manual correction of the obtained data resulted in 46 gene clusters related to secondary metabolism (Table 4). This makes us think that the *K. albida* genome is one of the richest in terms of secondary metabolism genes reported till now. General features of the secondary metabolism gene clusters of *K. albida* are summarized in Additional file 1: Table S2.

The most represented type of secondary metabolite biosynthesis genes within the *K. albida* genome are non-ribosomal peptide synthases (NRPS) (16 out of 46; Table 4, Additional file 1: Table S2). Core genes in clusters *kal* 1, 9, 14, 16, 30, and 35 encode NRPS proteins with only three domains: adenylation (A), peptidyl-carrier protein (PCP), and an approximately 700 aa long N-terminal domain that is proposed to act as condensation domain (C) [49]. NRPS genes in these clusters are accompanied by genes usually found in the biosynthesis of siderophores [50–53]. Several other NRPS clusters in the genome could be designated as siderophores producing due to the presence of genes encoding ornithine N-monooxygenase (*kal22*) or isochorismate (salicylate) synthase (*kal27*, *kal32*, *kal44*) known to be involved in biosynthesis of precursors [52–54], or conserved Fe^3+^-siderophore ABC transport system genes (*kal27* and *kal44*) [55]. Besides NRPS produced siderophores, *kal23* cluster is predicted to be responsible for biosynthesis of the aerobactin-like Fe^3+^-chelating compound found in different species of *Corynebacterium* and *Bacillus* [56].

The second most abundant *K. albida* secondary metabolism genes are encoding polyketide biosynthesis. Analysis of the genome revealed 6 type I PKS (*kal 3, 6, 28, 39, 40,* and *46*) and 2 type II PKS (*kal21* and *45*) gene clusters (Table 4, Additional file 1: Table S2). The *kal3* cluster consists of one gene encoding single module PKS (KALB_1318) with a full set of ketoreductase domains. The closest homologues of this enzyme encode mycocerosic acid synthase in *Stigmatella aurantiaca* and in several *Mycobacterium* species involved in the production of the multimethyl-branched fatty acids by the elongation of fatty acids with 4 units of methyl-malonate [57, 58]. The core genes in the cluster *kal28* (KALB_5021, 5022, 5023) are similar to genes encoding omega-3-polyunsaturated fatty acid synthases. These unusual fatty acids are known to accumulate in the membranes of cold- and high pressure-resistant marine bacteria [59, 60]. Other type I PKS gene clusters (*kal 6, 39, 40* and *46*) have no homologues in another sequenced bacterial genomes and their products could be just partially predicted from the genes organization. Two other PKS clusters encode type II PKS systems that show some similarity to *whiE* clusters involved in spore pigment production in different streptomycetes (*kal21*) [61, 62] and to genes involved in biosynthesis of the aromatic polyketides mithramycin [63] and nogalamycin [64] (*kal45*).

Seven *K. albida* gene clusters combine type I PKS and NRPS biosynthetic pathways (Table 4, Additional file 1: Table S2) [65]. Gene clusters *kal4* and *kal10* encode type I PKSs and a NRPSs with a single condensation domain. The PKS portion of *kal10* resembles the KS involved in production of phenolic glycolipids in *Mycobacteria*, another Fe^3+^-chelating compound [66]. The NRPS part might act at the initiation step by direct loading of the starting unit into the ACP of PKS, similar to the mechanism proposed for biosynthesis of mycobactins, mycobacterial siderophores of the phenolic glycolipids family [67, 68].

5 gene clusters for terpenoid biosynthesis could be found in the *K. albida* genome (Table 4, Additional file 1: Table S2). The C5 precursors for these secondary metabolites are provided by the non-mevalonate (MEV/DOXP) pathway, genes for which are present in the genome [69, 70]. At the same time we were unable to identify any genes encoding enzymes of the mevalonate pathway. The *kal2* and *kal25* gene clusters a predicted to be involved in the production of the earthy flavored sesquiterpene geosmin and moldy-smelling monoterpene 2-methylisoborneol (2-MIB) respectively [71, 72]. Core genes of both of them are highly conserved among different actinomycetes. Interestingly, there is one more germacradienol synthase gene (KALB_0677, *geo2*) located upstream from *geo1* (KALB_0676, *geo1*) in *kal2* cluster. Its function is not clear. *kal5* cluster is predicted to be involved in the biosynthesis of carotenoids based on the key gene *CtrB5* (KALB_2031) similarity to the known phytoene synthases [73]. Similar, *kal20* gene cluster is suggested to be involved in the biosynthesis of hopanoids, bacterial pentacyclic triterpenoids [74].

Several other types of secondary metabolites could be potentially produced by *K. albida* (Table 4, Additional file 1: Table S2). The gene cluster *kal8* encodes 4 enzymes involved in the biosynthesis of compatible solutes ectoine and 5-hydroxyectoine [75, 76]. The *kal11* cluster is similar to genes involved in biosynthesis of indolocarbazole group of secondary metabolites [77, 78]. Several lantibiotics biosynthesis gene clusters could be found in the genome of *K. albida* as well (*kal12, 13, 29, 41, 42)*. Interestingly, ORFs 3600 and 3601 from *kal13* are coding for YcaO domain-containing proteins involved in the formation of thiazole/oxazole, another post-translational modifications observed in ribosomally synthesized natural products [79, 80]. The cluster *kal24* contains only one gene (KALB_4567), which encodes a 13 kDa protein with the high degrees of similarity to bacteriocins of the linocin M18 family [81]. Linocin M18 was first isolated from *Brevibacterium linens* due to its ability to inhibit the growth of several *Listeria* species, and similar genes were later discovered in other bacterial genomes. Recent findings indicate that linocins might play a role in the compartmentalization of oxidative-stress response processes in bacterial cells [82]. At the same time, the product of KALB_4567 is two times shorter than typical linocin M18.

### Testing secondary metabolism potential

The genome sequence of *K. albida* unveiled enormous secondary metabolites biosynthetic potential of this bacterium (Additional file 1: Table S2). However, so far only aculeximycin is known to be produced by this strain [11, 12]. To further elucidate the biosynthetic potential of *K. albida* the strain was grown in different media and extra- and intra-cellular accumulated metabolites were tested using high resolution LC-MS (Additional file 1: Figures S1, S2). After 7 days of growth aculeximycin and its aglycone production was observed only in two out of six used media. At the same time, multiple secondary metabolites accumulation was observed in all cases. The DNP analysis [83] of obtained data led to idea that majority of compounds accumulated by *K. albida* are not described yet. Furthermore, extracts were found to be active against *Bacillus subtilis*, even those that did not contain aculeximycin. These facts are making further analysis of secondary metabolites produced by *K. albida* especially interesting.

As expected from the secondary metabolism genes analysis many of the metabolites produced by *K. albida* are acting as siderophores (Additional file 1: Table S2; Additional file 1: Figure S3). A siderophores accumulation test using a modified CAS assay clearly showed that *K. albida* produced significant amounts of Fe^3+^-chelating compounds during growth on different media when compared to *S. coelicolor* and *S. albus* (Additional file 1: Figure S3) [84]. Additionally, extracts from cultures grown on tested media including liquid and solid CAS media were separated by TLC and overlaid with CAS agarose for siderophores detection (Additional file 1: Figure S3). As a result, *K. albida* was found to accumulate from one to at least 5 Fe^3+^-chelating compounds with different physicochemical properties when growing at different conditions. Any of them could be predicted based on information available in DNP [83]. This finding supports our prediction of evolving of the *K. albida* genome in directions leading to adaptation to iron deficient environment, where the strain was isolated from [6, 12].

**Figure 4:**
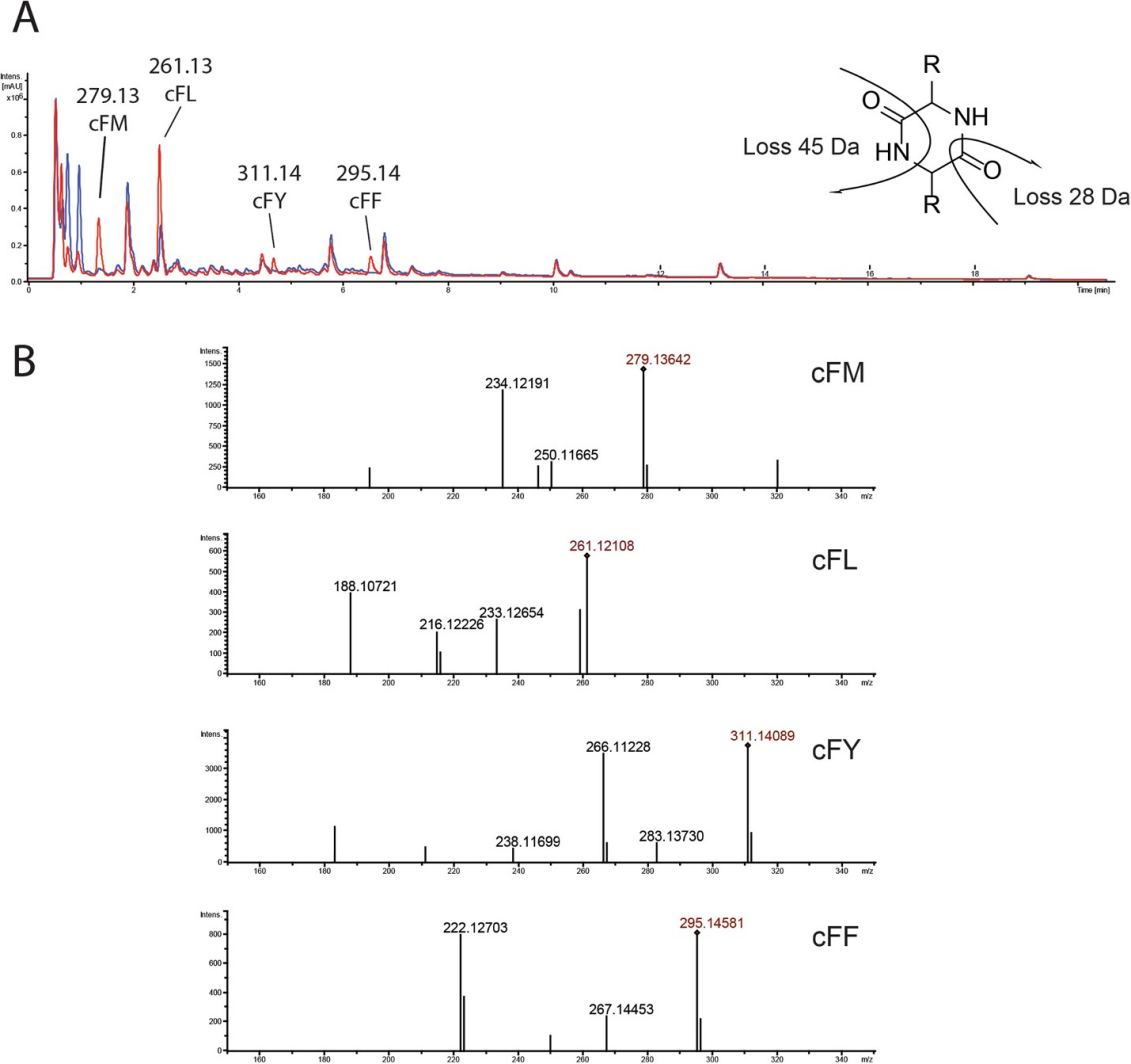
**HPLC-MS analysis of extracts from** ***E. coli*** **expressing KALB_7471. A**. UV–vis traces that correspond to the KALB_7471 expressing strain (red) and control strain (blue) are shown. Cyclic dipeptides are marked and their masses are indicated. **B**. MS2-fragmentation spectra of KALB_7471 products. The characteristic neutral losses of 28 and 45 Da, resulting in the detection of the respective ammonium ions are shown.

One of the previously described compounds that were identified within the extracts from *K. albida* is a cyclic leucilphenylalanine (cFL) (Additional file 1: Figure S2). It was produced in 4 out of 7 tested media. This compound accumulation could be directly linked to the cyclodipeptide synthase gene (KALB_7471) found in the *kal43* cluster. These proteins comprise an interesting class of enzymes utilizing amino-acyl tRNA as a substrate to produce diketopiperazine containing cyclic dipeptides [85–87]. In many cases the cyclodipeptide synthase products are further modified by the decorating enzymes. No genes possibly involved in post-processing of cyclic peptides were found in close proximity to the KALB_7471. The number of recently discovered cyclodipeptide synthases is expanding. In many cases these enzymes are not strictly specific for some particular substrates and can produce several types of cyclic peptides [85, 87]. However, we were not able to identify any other possible products of KALB_7471 in extracts of *K. albida*. In order to test the enzyme specificity the gene was cloned and expressed in *E. coli*. Comparison of extracts from *E. coli* containing KALB_7471 expression construct and empty vector control led to identification of four new compounds (Figure 4). One of them, as was predicted from analysis of *K. albida* extracts, was cFL. Three other compounds were identified as cFM, cFY and cFF based on exact mass and fragmentation patterns (Figure 4). This finding led us to the conclusion that the *K. albida* enzyme as the first substrate prefers phenylalanine, but can also utilize other amino acids as a second substrate. Re-examination of *K. albida* extracts led to identification of cFF and cFY, however not cFM.

## Conclusions

The complete genome of *Kutzneria albida*, the first representative of *Kutzneria* genus was sequenced, annotated and analyzed. The genome of this strain is one of the biggest circular actinobacterial genomes sequenced thus far. The phylogenetic and orthology analyses clearly distinguish *Kutzneria* albida from *Streptosporangiaceae* thereby providing the first genomic evidence for transferring the genus into the *Pseudonocardiaceae* family.

Two large genomic islands are present in the *K. albida* genome. Localization of these islands corresponds to regions of a high density of genes involved in secondary metabolism providing clues into the origin of a large part of the strain’s auxiliary metabolism. In general, about 14% of the chromosome is occupied with the secondary metabolism gene clusters, including cluster predicted to be involved in aculeximycin biosynthesis.

Aculeximycin and its aglycone accumulation were observed during growth of the strain in several media. However, a vast majority of compounds produced by the strain were not found in available secondary metabolites databases. As predicted from the genome analysis and confirmed experimentally, a large proportion of secondary metabolism of *K. albida* is devoted to siderophores production. On the other hand, cyclic dipeptides were found in the extract of the strain.

In summary, sequencing of the *K. albida* genome provides new insights into understanding the evolution of minor groups of actinobacteria and will attract more attention to these fascinating bacteria as an inexhaustible source of novel biologically active secondary metabolites. The large diversity of secondary metabolism gene clusters in the genome of *K. albida* is reflected in metabolites produced. Furthermore, isolation, structural and biological characterization of secondary metabolites produced by this strain might lead to discovery of new interesting biological activities as well as new chemical scaffolds thus proving the concept of genome mining of minor groups of actinobacteria for new secondary metabolites discovery.

## Methods

### Sequencing of *Kutzneria albida*genome

The type strain of *Kutzneria albida* (DSM 43870^T^) was obtained as a lyophilized culture from DSMZ (Braunschweig, Germany). Genomic DNA was isolated from 30 ml cultures grown in tryptone soy broth (TSB) [88] at 28°C for 24 hours. Total DNA isolation was performed according to the salting out procedure followed by RNase treatment [88]. The obtained DNA was used to construct both a 12 k PE and a WGS library for pyrosequencing on a Genome Sequencer FLX (Roche Applied Science), using the Titanium chemistry to reduce problems with high G + C regions [89]. Assembly of the shotgun reads was performed with the GS Assembler software (version 2.3). A total of 491,980 reads (170,469,390 bp) were assembled into 197 contigs in 1 scaffold.

### Completion of the draft sequence

For finishing of the genome sequence, the CONSED software package was used [90]. Of the 197 gaps, 57 could be closed in silico as these gaps were caused by repetitive elements. For gap closure and assembly validation, the remaining genomic contigs were bridged by 140 PCR products.

Gaps between contigs of the whole genome shotgun assembly were closed by sequencing PCR products carried out by IIT GmbH (Bielefeld, Germany) on ABI 377 sequencing machines. To obtain a high quality genome sequence and to correct for homopolymer errors common in pyrosequencing, additional Illumina GAIIx data was used. A total of 5,064,677 reads of 50 bp length was mapped on the genome, resulting in a 25.6x coverage. A total of 19 SNPs, 50 single nucleotide insertions and 49 single nucleotide deletions were found and corrected.

### Genome analysis and annotation

In the first step, gene finding was done using GISMO [91] followed by GenDB 2.0 automatic annotation [92]. In the second annotation step, all predicted ORFs were manually re-inspected to correct start codon and function assignments. Intergenic regions were checked for ORFs missed by the automatic annotation using BLAST [93].

### Phylogenetic analysis

19 rDNA sequences were aligned using MAFFT v7.017 (gap open penalty 1.53, offset value 0.123, scoring matrix 200PAM/k = 2, algorithm: auto). Dendrogram was built using Geneious [94] (Tamura-Nei genetic distance model, neighbor-joining method, *E. coli* as an outgroup, bootstrap value 1000, consensus tree with 50% support threshold). Ribosomal RNA sequences for all strains but *S. albus* were obtained from the Silva rRNA database [32].

### Orthology analysis

10 genomes were used for the analysis of the numbers of orthologous genes between genome pairs (GenBank accession version is indicated in parenthesis): *Actinosynnema mirum* DSM 43827^T^ (CP001630.1), *Amycolatopsis mediterranei* S699 (CP003729.1), *Kitasatospora setae* NBRC 14216^T^ (AP010968.1), *Kutzneria albida* DSM 43870^T^ (deposited to GenBank with accession CP007155), *Saccharopolyspora erythraea* NRRL 2338 (AM420293.1), *Saccharotrix espanaensis* DSM 44229^T^ (HE804045.1), *Streptomyces avermitilis* MA-4680 (BA000030.3), *Streptomyces coelicolor* A3(2) (AL645882.2), *Streptomyces griseus subsp. griseus* NBRC 13350 (AP009493.1), *Streptosporangium roseum* DSM 43021^T^ (CP001814.1). In the first step, 45 pairwise genome-wide reciprocal best-hit protein BLAST searches were performed on 10 genomes, using InParanoid [95] (configured as follows: two-pass BLAST, with bootstrapping, not using an outgroup, matrix BLOSUM45, minimal BLAST bit score 40, sequence overlap cut-off 0.5, segment coverage overlap 0.25). Pseudo genes were excluded from this step. In the next step, MultiParanoid [96] was applied (with default parameters – genes clustered twice were not removed) to generate single file of orthologous gene clusters. The file contains a total of 8,745 orthologous gene clusters with 65,033 genes. Finally, this file was parsed, returning – for each analyzed pair of genomes – the number of orthologous gene clusters which contained genes from both of these genomes. This number was then reported. To find the number of genes common to all 10 genomes, we identified the number of orthologous gene clusters containing 10 unique genome identifiers.

### Analysis of secondary metabolite clusters

For the identification of secondary metabolite clusters, the genome of K. albida was scanned for homologues to known secondary metabolite synthases via BLAST search. These manual investigations were supported by antiSMASH [29]. A set of genes was considered to be a cluster, when there was at least one gene encoding a secondary metabolite synthase. Consequently, a locus possessing a gene with only a single domain, for example an A domain, was not considered to be a cluster. The boundaries of the clusters were defined by the last gene upstream and downstream of a secondary metabolite synthase with homology to a gene encoding a regulator, transporter or tailoring enzyme. In cases where this gene was part of a putative operon, the whole operon was included into the cluster. The modular organization of the type I polyketide and nonribosomal peptide megasynthases were determined using web tools [97, 98].

### Secondary metabolites production and LC-MS analysis

*K. albida* was grown in 20 ml of TSB media for 4 days. 2 ml of pre-culture was inoculated into 50 ml of production media. Six different medias were used: TSB, NL5 (NaCl 1 g/l, KH_2_PO_4_ 1 g/l, MgSO_4_x7H_2_O 0.5 g/l, Trace elements solution 2 ml/l, Glycerol 25 g/l, L-glutamine 5.84 g/l), NL19 (Soy flour 20 g/l, Mannitol 20 g/l), NL111 (Meat extract 20 g/l, Maltose extract 10 g/l, CaCO_3_ 10 g/l), CAS [84] and SG [99]. Strain was grown for 7 days at 30°C and 250 rpm. Metabolites were extracted with ethyl acetate from supernatant and acetone-methanol (1:1) mixture from biomass. Extracts were evaporated, dissolved in 100 μl of methanol and samples from biomass and supernatant were combined. 1 μl of each sample were separated on an Ultimate 3000 HPLC (Dionex) using C18 column (Affymetrix) and linear gradient of acetonitrile against 0.1% ammonium formate solution in water. Samples were analyzed on ultrahigh resolution mass spectrometer system maXis (Bruker Daltonics).

### Cloning and expression of KALB_7471

KALB_7471 was amplified from the genome of *K. albida* using KOD polymerase (Novagen) and primers Kal7474LETNcF (TACCATGGTGTTGACCAC GAGCCCATT) and Kal7474ETBR (CGGATCCCGCACGGCCAGCGATTCGG) and cloned as *Nco*I/*BamH*I into pET28b. Obtained plasmid was introduced into *E. coli* BL21(DE3). Strain was grown in 50 ml of LB media till OD600 0.4 and expression was induced with 0.5 mM IPTG. Accumulation of KALB_7471 protein was tested after 8 hours of growth at 30°C by SDS PAGE. Metabolites were extracted with ethyl acetate, evaporated, dissolved in 200 μl of methanol and analyzed as described above. *E. coli* BL21(DE3) containing empty pET28b was used as control.

### Nucleotide sequence accession numbers

The genome of *Kutzneria albida* was deposited to GenBank with accession number CP007155.

### Supporting information

Data sets supporting the results of this article are included within the article and its Additional file 1.

## Electronic supplementary material

Additional file 1: Table S1: Annotation and prediction of gene functions of aculeximycin biosynthesis gene cluster. Locus number is the numeric part of the locus_tag (e.g. locus number 6558 refers to gene KALB_6558). **Table S2.** Classification and characterization of secondary metabolism (SM) gene clusters identified in *Kutzneria albida* DSM43870 genome. **Figure S1.** HPLC-MS analysis of extracts from *K. albida* culture grown in different media. UV–vis traces are shown. Masses of some compound are marked. Compounds that have hits in DNP are also indicated. **Figure S2.** HPLC-MS analysis of extracts from *K. albida* culture grown in different media. Base peak chromatogram traces are shown. Masses of some compound are marked. Aculeximycin and its aglycone are marked as Acu and AcuA respectively. Cyclic dipeptides are marked as T1 (cFL), T2 (cFY) and T3 (cFF). **Figure S3.** A. Modified CAS siderophore production test. Test was performed as described in [1]. Change in color was monitored after 3 hours. Orange color indicates siderophores accumulation. B. TLC analysis of extracts from K. albida cultures grown in different media (1 – TSB supernatant, 2 – TSB biomass, 3 – NL5 supernatant, 4 – NL5 biomass, 5 – NL19 supernatant, 6 – NL19 biomass, 7 – NL111 supernatant, 8 – NL111 biomass, 9 – CAS supernatant, 10 – CAS biomass, 11 – SG supernatant). The solvent phase was acetone-methanol 9:1. Plate was overlaid with 0.8% CAS agar. Changes in color were monitored after 30 minutes of incubation. (PDF 1 MB)
