# Supplementary material for: Complete genome sequence of producer of the glycopeptide antibiotic Aculeximycin Kutzneria albida DSM 43870T, a representative of minor genus of Pseudonocardiaceae
Source: BMC Genomics. 2014 Oct 10;15(1):885. doi: 10.1186/1471-2164-15-885 (PMC4210621; doi:10.1186/1471-2164-15-885)
Supplement: Supplementary file 1 — Additional file 1: Table S1: Annotation and prediction of gene functions of aculeximycin biosynthesis gene cluster. Locus number is the numeric part of the locus_tag (e.g. locus number 6558 refers to gene KALB_6558). Table S2. Classification and characterization of secondary metabolism (SM) gene clusters identified in Kutzneria albida DSM43870 genome. Figure S1. HPLC-MS analysis of extracts from K. albida culture grown in different media. UV–vis traces are shown. Masses of some compound are marked. Compounds that have hits in DNP are also indicated. Figure S2. HPLC-MS analysis of extracts from K. albida culture grown in different media. Base peak chromatogram traces are shown. Masses of some compound are marked. Aculeximycin and its aglycone are marked as Acu and AcuA respectively. Cyclic dipeptides are marked as T1 (cFL), T2 (cFY) and T3 (cFF). Figure S3. A. Modified CAS siderophore production test. Test was performed as described in [1]. Change in color was monitored after 3 hours. Orange color indicates siderophores accumulation. B. TLC analysis of extracts from K. albida cultures grown in different media (1 – TSB supernatant, 2 – TSB biomass, 3 – NL5 supernatant, 4 – NL5 biomass, 5 – NL19 supernatant, 6 – NL19 biomass, 7 – NL111 supernatant, 8 – NL111 biomass, 9 – CAS supernatant, 10 – CAS biomass, 11 – SG supernatant). The solvent phase was acetone-methanol 9:1. Plate was overlaid with 0.8% CAS agar. Changes in color were monitored after 30 minutes of incubation. (PDF 1 MB) [file 12864_2014_6593_MOESM1_ESM.pdf]

Table S1. Annotation and prediction of gene functions of aculeximycin biosynthesis gene cluster. Locus number is the numeric part of the locus_tag (e.g. locus number 6558 refers to gene KALB_6558).

Gene name	Locus number	Direction	Location start	Location end	Product
acuX1	6558	reverse	7364671	7362971	Hypothetical protein
acuGT8	6559	reverse	7366220	7365003	Glycosyltransferase
acuAVIII	6560	reverse	7377376	7366235	PKSI
acuAVII	6561	reverse	7386327	7377424	PKSI
acuAVI	6562	reverse	7409882	7386381	PKSI
acuAV	6563	reverse	7423160	7409934	PKSI
acuAIV	6564	reverse	7432829	7423185	PKSI
acuAIII	6565	reverse	7438114	7432850	PKSI
acuAII	6566	reverse	7453128	7438144	PKSI
acuAI	6567	reverse	7469754	7453189	PKSI
acuO2	6568	reverse	7471178	7469967	Cytochrome P450 hydroxylase
acuGT7	6569	reverse	7472406	7471171	Glycosyl transferase
acuGT6	6570	reverse	7473617	7472403	Glycosyltransferase
acuS6	6571	reverse	7474507	7473641	Glucose-1-phosphate thymidylyltransferase
acuW	6572	reverse	7476426	7474630	ABC Transporter protein
acuR5	6573	forward	7476529	7477119	Transcription regulator, TetR family
acuGT5	6574	forward	7477412	7478632	Glycosyltransferase
acuGT4	6575	reverse	7480109	7478919	Glycosyltransferase
acuS5	6576	reverse	7480929	7480129	NDP-hexose-4-ketoreductase
acuH	6577	reverse	7482659	7481049	Beta-hexosaminidase
acuN2	6578	forward	7482946	7484181	Aminotransferase
acuGT3	6579	forward	7484178	7485428	Antibiotic resistance macrolide glycosyltransferase
acuN1	6580	forward	7485514	7486623	Aminotransferase
acuGT2	6581	forward	7486701	7487948	Glycosyltransferase
acuS4	6582	reverse	7489725	7488259	NDP-hexose 2,3-dehydratase
acuS3	6583	reverse	7490428	7489823	dTDP-4-dehydrorhamnose 3,5-epimerase
acuGT1	6584	forward	7490555	7491754	Glycosyltransferase
acuS2	6585	forward	7491783	7492757	dTDP-glucose 4,6-dehydratase
acuR4	6586	reverse	7493563	7492823	LuxR-family transcription regulator
acuO1	6587	forward	7494079	7494945	F420 Monooxygenase

acuS1	6588	reverse	7496334	7495132	NDP-hexose-C3-methyltransferase
acuR3	6589	reverse	7499231	7496448	LuxR-family transcription regulator
acuR2	6590	reverse	7501849	7499231	LuxR-family transcription regulator
acuR1	6591	reverse	7504512	7501846	LuxR-family transcription regulator

Table S2. Classification and characterization of secondary metabolism (SM) gene clusters identified in *Kutzneria albida* DSM43870 genome. Abbreviations of NRPS and PKSI domains: C – condensation, A – adenylation, PCP – peptidyl-carrier protein, E – epimerase domain, nMT – N-methyltransferase, TE – thioesterase, KS – ketosynthase, AT – acyltransferase, ACP – acyl carrying protein, KR – ketoreductase, DH – dehydratase, ER – enoylreductase, AmT – aminotransferase, SDR – short chain dehydrogenase/reductase.

Type of SM	Cluster	Core enzymes locus tag and their domains architecture	Modification enzymes
		**NRPS**	
	kal7	2585: A,PCP<br>2589: C,A,PCP,C,A,PCP,E,C,A,PCP,E,C,A,PCP,C,A,E,PCP<br>2590: C,A,PCP,C,A,PCP,E<br>2591: C,A,nMT,PCP,E,C,A,PCP,E,C,A,PCP,TE	Reductase (2586), oxygenase (2593), halogenase (2594), SARP transcriptional regulator (2597)
	kal17	3899: C,A,PCP;C,A,nMT,PCP,TE	ER (3875), reductase (3876),
	kal18	3904: A,PCP;C,A,PCP;E,C,A,PCP;C,PCP;C,A,PCP;C,PCP;C,A,PCP;C,A,PCP;C,A,PCP,E	LuxR transcriptional regulators (3906, 3908), cytochromes P450 (3912), SDR (3919)
	kal36	6133: PCP,C,A,PCP;C,A,PCP,TE<br>6134: A<br>6137: A,PCP;E,C,A,PCP;C,A,PCP,C	SDR (6127), dioxygenase (6139), SARP transcriptional regulator (6144)
	kal37	6150: A,PCP,TE<br>6152: A,KR,PCP<br>6154: C,A,PCP;C,A,PCP;C,A,PCP,TE<br>6155: A,PCP;C,PCP;E,C,PCP;C,A,PCP;C,PCP;C,A,PCP;C,A,PCP;C,A,PCP,E<br>6159: TE	ornithine cyclodeaminase (6148), dioxygenases (6149, 6160), amidase (6156), Ornithine transcarbamoylase (6158), glycosyltransferase (6172)
	kal38	6220: C,PCP<br>6221: A<br>6225: PCP<br>6227: A<br>6233: TE<br>6234: C,A PCP<br>6235: PCP<br>6240: A	dehydrogenases (6199, 6214, 6223), SDR (6201, 6232), phosphopantetheinyl transferase (6222), oxygenase , DAP diaminopimelate decarboxylase (6226), N-methyltransferase (6238)
		**Siderophore NRPS**	

	kal1	0436: A,PCP, 700 aa N-terminal domain	Aminotransferase (0437)
	kal9	3278: A, PCP, 700 aa N-terminal domain	
	kal14	3665: A,PCP, 700 aa N-terminal domain	Aminotransferase (3666)
	kal16	3809: A,PCP, N-terminal catalytic domain (ε-poly-L-lysin synthases)	isochorismatase (3803)
	kal22	4308: A,PCP,C,PCP<br>4312: TE	Aminotransferase (4299), ornithine N-monooxygenase (4309), SDR (4301).
	kal27	4803: PCP<br>4805:A<br>4810: C,A,PCP;E,C,A,PCP,E<br>4811: C,A,PCP;C,A,PCP;C,A,PCP;E,C,A,PCP<br>4812: C,A,PCP;C,A,PCP;E,C,A,PCP;E,C,A,PCP,E	dioxygenases (6789), isochorismatase (4803, 4804), isochorismate synthase (4806), SDR (4808), iron ABC transporter (4809)
	kal30	5129: A,PCP, 700 aa N-terminal domain	Oxidoreductase (5135)
	kal32	5550: A<br>5551: C	Isochorismate synthase (5549), oxidoreductase (5552)
	kal35	5976: A,PCP, 700 aa N-terminal domain	
	kal44	7696: C,A,PCP,TD<br>7697: PCP<br>7698: A	Aminotransferase (7683), SDR (7687), isochorismatase (7689), iron ABC transporter (7693-7695), dioxygenases (7700)
		**PKS**	
Type I	*kal3*	1318: KS,AT,DH,ER,KR,ACP	ER (1311), KR (1313), epimerase (1319)
Type I	*kal6*	2529:KS,AT,ACP;KS,AT,KR,ACP;KS,AT,KR,ACP	KR (2525), ER (2526)
Type II	*kal21*	4117:KSα<br>4118:KSβ<br>4114:ACP	cyclases (4113, 4115), oxygenases (4119), cytochromes P450 (4121)
**Type I (IV)**	*kal28*	5021: KS,AT,DH,DH,ACP<br>5022: KR<br>5023: KS,AT,ACP<br>5025: ACP	Aminotransferase (5014), dehydrogenases (5018, 5019, 5026), ACP-malonyltransferase (5020), TE (5024)

**Type I**	*kal39*	6321: ACP<br>6322: AT,DH<br>6323: KS,AT,KR	Glycosyltransferase (6320), SDR (6325)
Type I<br>**Acculeximycin biosynthesis gene cluster**	*kal40*	6567 *acuI*: KS,AT,DH,ER,KR,ACP;KS,AT,KR,ACP;KS,AT,DH,ER,KR,ACP<br>6566 acuII: KS,AT,KR,ACP;KS,AT,DH,ER,KR,ACP;KS,AT,KR,ACP<br>6565 *acuIII*: KS,AT,DH,KR,ACP;<br>6564 *acuIV*: KS,AT,DH,KR ACP;KS,AT,KR, ACP<br>6563 *acuV*: KS,AT,KR,ACP;KS,AT,KR, ACP;KS,AT,KR,ACP;<br>6562 *acuVI*: KS,AT,KR,ACP;KS,AT,KR,ACP;KS,AT,DH,ACP;KS,AT,KR,ACP;KS,AT,DH,ER,KR,ACP<br>6561 *acuVII*: KS,AT,KR, ACP;KS,AT,KR, ACP<br>6560 *acuVIII*: KS,AT,DH,KR,ACP;KS,AT,DH,KR,ACP;TE	
Type II	*kal45*	7829: ACP<br>7830: KSβ<br>7831: KSα	cyclases (7824), oxygenases (7826,7834,7839), SDR (7810,7822,7825,7833), glycosyltransferases (7812,7813,7814), O-methyltransferase (7835)
Type I	*kal46*	8454: KS,AT,KR<br>8455: KS,AT<br>8456: ACP	Aminotransferase (8447), glycosyltransferases (8458), cyclase (8466)
		**PKS-NRPS hybrid**	
	kal4	1470:KS,AT,DH,KR,ACP;KS,AT,DH,KR,ACP<br>1471:KS,AT,KR,ACP<br>1472:KS,AT,DH,KR,ACP;KS,AT,DH,KR,ACP;KS,AT,DH,KR,ACP<br>1473:KS,AT,DH,KR,ACP,TE<br>1477: AT<br>1478:ACP<br>1479:A<br>1480:A<br>1483:ACP,KS,AT,DH,KR,ACP;KS,AT,DH,KR,ACP;KS,AT,DH,KR,ACP<br>1488:C,A	Cyclase, cytochromes P450 (1468,1475), glycosyltransferase (1474), diaminopimelate decarboxylase (1481)
	kal10	3293:C<br>3305: Acyl-CoA ligase<br>3306:ACP,KS,AT,DH,KR,ACP,TE	Cytochrome P450 (3295), dehydrogenase (3301), glycosyltransferase (3316)

	kal19	3936: A,PCP;C,A,PCP;E,C,A,PCP;E,C,A,PCP;E,C,A,PCP<br>3937: C,A,PCP;E,C,A,PCP;C,A,PCP;E,C,A,PCP;C,A,PCP,E<br>3948: ACP<br>3951: Acyl-CoA ligase<br>3952: AT,ACP,KS,AT,KR,ACP<br>3953: KS,AT,DH,KR,ACP,TE	SDR (3919), methyltransferase (3934,3947), cytochromes P450 (3944,3945)
	kal26	4737: ACP,KS,AT,ACP,AmT,C,A,PCP,C,A,PCP<br>4740: Acyl-CoA ligase<br>4741: C,A,nMT,PCP,C<br>4742: C,A,PCP,TE<br>4759: A,PCP, terminal dehydrogenase	KR (4725), dehydrogenase (4726), ER (4746)
	kal31	5510: A,PCP<br>5511: KS<br>5512: A,PCP;C,PCP;C,A,PCP;C,A,PCP;C,A,PCP,TE<br>5522: A,PCP	Tyrosine 2,3-aminomutase (5509), methyltransferase (5514), dehydrogenase (5515), ornithine cyclodeaminase (5516)
	kal33	5790:A<br>5798: KS,ACP;C,A,PCP,TE<br>5802:A,PCP<br>5807:A	monooxygenase (5788), methyltransferase (5789), KR (5791), glycosyltransferases (5799, 5805), cytochromes P450 (5792,5800,5804), SDR (5809)
	kal34	5887: KS<br>5890: KS<br>5893: KS<br>5905: C,A,PCP;E,C,A,PCP;C,A,PCP,TE<br>5924:A, PCP;C,A,PCP<br>5925:C	methyltransferase (5884), SDR (5887), halogenase (5897), cytochromes P450 (5903), dioxygenases (5896,5911), aminotransferase (5917)
		**Terpene**	
Geosmin	*kal2*	0676, geo1: germacradienol-geosmin synthase<br>0677, geo2: germacradienol synthase	Geranyl-geranyl-pyrophosphate synthetase (0679)
	kal5	2031 crtB5:phytoene synthase<br>2032crtX: carotene biosynthesis associated membrane protein	Geranyl-geranyl-pyrophosphate synthase (KALB_6731, ispA5), phytoene desaturase (KALB_6732, crtI5)
	kal15	3743: *crtL* type lycopene cyclase gene	

	kal20	4042: radical SAM protein<br>4044: squalene-hopene cyclase<br>4045: trans-Isoprenyl Diphosphate Synthases<br>4046: squalene-associated FAD-dependent desaturase<br>4047: phytoene synthase<br>4048: phytoene synthase	SDR (4040,4057), glycosyltransferases (4037), 4-Hydroxy-3-methylbut-2-enyl diphosphate reductase (4043)
2-MIB	*kal25*	4603: cyclo-AMP binding protein<br>4604: SAM dependent GPP methyltransferase<br>4605: monoterpene synthase	
		**Others**	
Ectoine	*kal8*	3094: Ectoine hydroxylase<br>3095: L-ectoine synthase<br>3096: Diaminobutyrate-pyruvate aminotransferase<br>3097: acetyl-transferase	Transporter (3098)
Indolocarbazole	*kal11*	3408: tryptophan oxidase<br>3409: chromopyrrolic acid synthase<br>3410: flavin monooxygenase	cytochromes P450 (3411,3412),acyl-CoA dehydrogenase (3413,3414), SDR (3416,3417,3418)
Lantipeptide	*kal12*	3444: LanM type lanthionine synthetase<br>3445: putative prepropeptide	Acyl-CoA ligase (3448)
Lantipeptide	*kal13*	*lanA13* (3595): prepropeptide<br>*lanB13* (3596): lanthionine-forming dehydratases<br>*lanC13a* (3597): lanthionine-forming dehydratases<br>*lanC13b* (3598): lanthionine-forming dehydratases<br>3599: peptide reductase/dehydrogenase<br>3600: thiazole/oxazole cyclodehydratase YcaO-like<br>3601: thiazole/oxazole cyclodehydratase YcaO-like	3588 - PKS: KS,AT,AmT
Aerobactin siderophore	*kal23*	4486: IucA/IucC domains protein	Transporter (4487), SDR (4488)
Bacteriocins	*kal24*	4567: linocin M18	ABC transporter (4568,4569)
Lantipeptide	*kal29*	5094: C,A (NRPS)<br>5095: LanM type lanthionine cyclase<br>5096: prepropeptide	Cyclase (5089), ER (5099),KR (5108), A-domain protein (5110)

Lantipeptide	*kal41*	6952: SpaB dehydratase, C-terminal domain protein<br>6953: Lantibiotic dehydratase<br>6954: prepropeptide<br>6955: LanC lanthionine cyclase	Methyltrasferase (6948)
Lantipeptide	*kal42*	7175: LanC lanthionine cyclase<br>7176: LanB dehydratase<br>7177: prepropeptide<br>7167: thiazole/oxazole cyclodehydratase YcaO-like	
Diketopiperazine	*kal43*	7471: cyclodipeptide synthase	

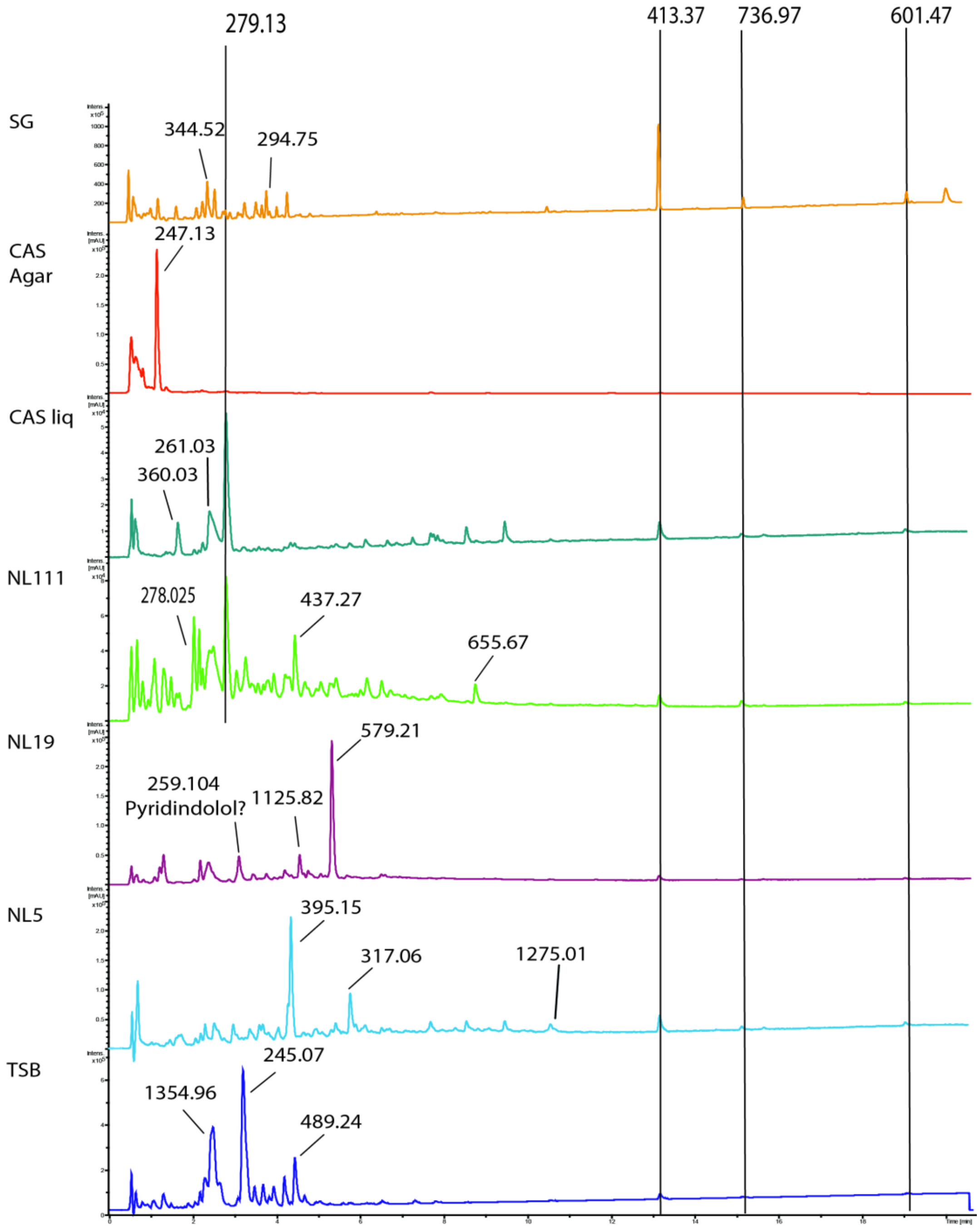

Figure S1. HPLC-MS analysis of extracts from *K. albida* culture grown in different media. UV-Vis traces are shown. Masses of some compound are marked. Compounds that have hits in DNP are also indicated.

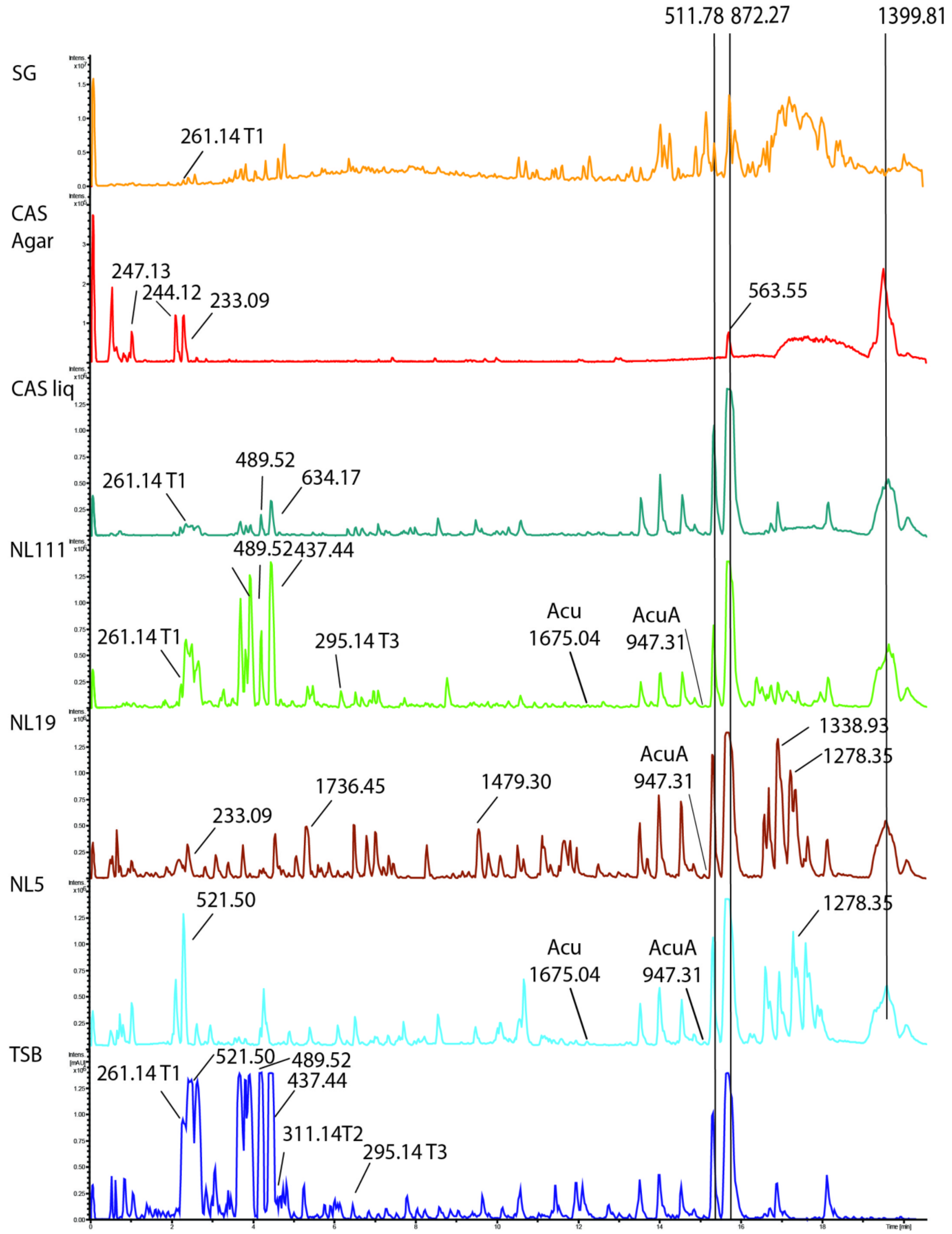

Figure S2. HPLC-MS analysis of extracts from *K. albida* culture grown in different media. Base peak chromatogram traces are shown. Masses of some compound are marked. Aculeximycin and its aglycone are marked as Acu and AcuA respectively. Cyclic dipeptides are marked as T1 (cFL), T2 (cFY) and T3 (cFF).

A

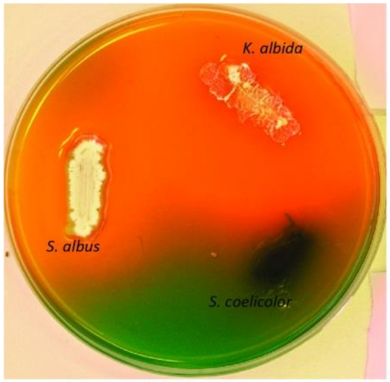

B

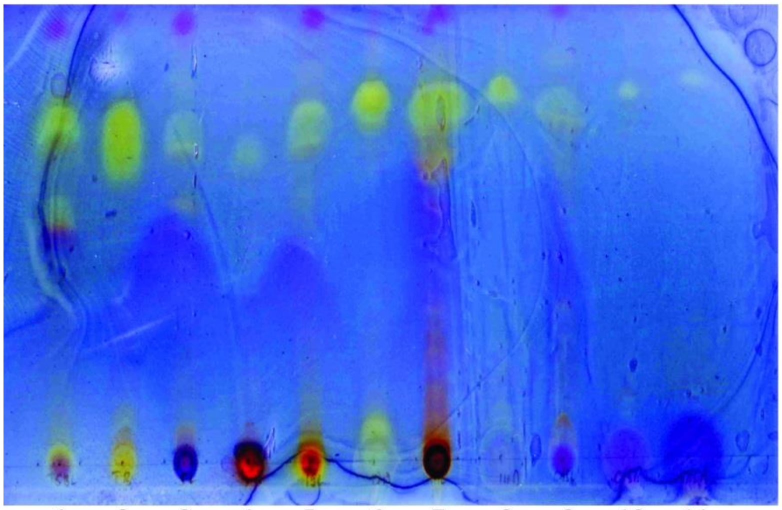

Figure S3. A. Modified CAS siderophore production test. Strains were grown for 7 days on CAS agar media and plates were overlaid with 0.8% agar containing Chrome azurol S (CAS), hexadecyltrimetyl ammonium bromide (HDTMA) in PIPES, and 1 mM$FeCl_3{\cdot}6H_2O$ [1]. Change in color was monitored after 3 hours. Orange color indicates siderophores accumulation. B. TLC analysis of extracts from K. albida cultures grown in diferent media (1 – TSB supernatant, 2 – TSB biomass, 3 – NL5 supernatant, 4 – NL5 biomass, 5 – NL19 supernatant, 6 – NL19 biomass, 7 – NL111 supernatant, 8 – NL111 biomass, 9 – CAS supernatant, 10 – CAS biomass, 11 – SG supernatant). The solvent phase was acetone-methanol 9:1. Plate was overlaid with 0.8% agar containing Chrome azurol S (CAS), hexadecyltrimetyl ammonium bromide (HDTMA) in PIPES, and 1 mM$FeCl_3{\cdot}6H_2O$. Changes in color were monitored after 30 minutes of incubation.

1. Perez-Miranda S, Cabirol N, George-Tellez R, Zamudio-Rivera LS, Fernandez FJ: **O-CAS, a fast and universal method for siderophore detection**. *J Microbiol Methods* 2007, **70**(1):127-131.
